# Atrial Fibrillation with Heart Failure in a Case with Resistance to Thyroid Hormone Due to a Rare Thyroid Hormone Receptor β Gene Mutation

**DOI:** 10.3390/ijms232315241

**Published:** 2022-12-03

**Authors:** Huei-Pin Lai, Mei-Hsiu Chen

**Affiliations:** 1 Division of Endocrinology, Department of Internal Medicine, Far Eastern Memorial Hospital, New Taipei City 220, Taiwan; 2 Department of Biomedical Engineering, Ming Chuang University, Taoyuan City 333, Taiwan

**Keywords:** resistance to thyroid hormone, thyroid hormone receptor β gene mutation

## Abstract

Resistance to thyroid hormone (RTH) is a rare disease typically associated with elevated levels of thyroid hormones and non-suppressed thyroid stimulating hormones. The most common cause of RTH is thyroid hormone receptor β (*THRβ*) gene mutation. Most individuals with RTH are considered clinical euthyroid. We report a family with a rare heterozygous point mutation, c.959G>T, (p.R320L) of the *THRβ* gene. The proband developed atrial fibrillation and life-threatening heart failure with pulmonary edema, which was quite different from previously reported *THRβ* gene mutations. Considering the rareness of RTH and the heterogeneity of its phenotypes, our report allows for a better understanding of the manifestation and management of patients with RTH and *THRβ* gene mutation.

## 1. Introduction

Resistance to thyroid hormone (RTH) is a rare disease with impaired tissue responsiveness to thyroid hormones (TH). The prevalence rate is about 1/40,000 with limited studies [1]. TH regulates physiological processes via separated receptor genes, thyroid hormone receptor β (*THRβ)* gene and thyroid hormone receptor α (*THRα)* gene. There are three receptor isoforms (*THRα1*, *THRβ1*, and *THRβ2*) which are mainly expressed in different tissues (*THRβ2*: hypothalamus and pituitary; *THRβ1*: liver; *THRα1*: central nervous system, myocardium, skeletal muscle, gastrointestinal tract, white adipose tissue) [2]. Most reported RTH cases are related to *THRβ* gene mutation (termed RTHβ), and the others are related to the mutations in the *THRα* gene, TH transport, and metabolism genes [3]. The clinical and biochemical characteristics of RTHβ are determined by the predominant receptor subtypes or isoforms expressed in that target organ. Laboratory data usually shows persistent elevation of circulating free T3 and free T4 levels, along with non-suppressed serum thyroid-stimulating hormone (TSH). The most common type of gene inheritance is autosomal dominant, but there are few cases with autosomal recessive inheritance, and some sporadic cases have also been noted [4]. The *THRβ* gene mutant has a dominant negative effect. That is, the mutation gene product adversely affects the normal gene product. Thus, cases with heterozygous *THRβ* gene mutation present more severe disease symptoms as compared with those with homozygous mutation [5]. In this study, we present a family with a rare heterozygous point mutation, c.959G>T, (p.R320L) of the *THRβ* gene. Our proband developed atrial fibrillation with decompensated heart failure because he missed a follow-up.

## 2. Case Report

### 2.1. Ethical Approval

This study was approved by the Institutional Review Board of Far Eastern Memorial Hospital (111178-C). All participants provided written informed consent.

### 2.2. Bioinformatics, Next-Generation Sequencing (NGS), and Sanger Sequencing

Genomic DNA was extracted from the subjects’ peripheral blood using a QIAGEN Gentra Puregene Blood kit. The DNA samples were prepared using Illumina TruSeq DNA PCR-Free Sample Preparation Kit. Probes were designed from XGen^®^ Predesigned Gene Capture Pools to target the coding regions of *THRα* and *THRβ* genes. An NGS was performed using Illumina MiSeq Reagent Kit v3 on a MiSeq platform (Illumina Inc., San Diego, CA, USA). The Genome Aggregation Database (gnomAD) (http://gnomad.broadinstitute.org/, accessed on 1 October 2022) and ClinVar (https://www.ncbi.nlm.nih.gov/clinvar/, accessed on 1 October 2022) were used to check for variants. The American College of Medical Genetics and Genomics (ACMG) guidelines were followed for variants interpretation. The variants that met the criteria for pathogenic were confirmed with traditional Sanger sequencing.

### 2.3. Proband

**Figure 1:**
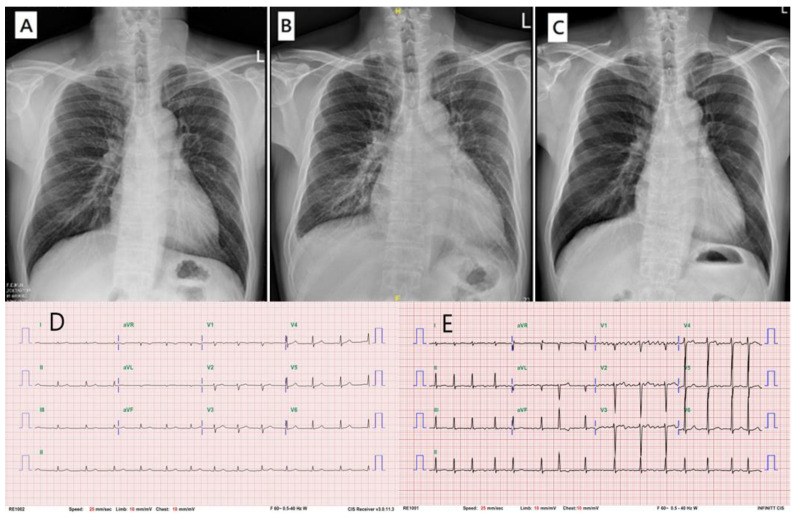
Cardiac complications of the proband. Initially, his chest X-ray (**A**) and EKG (**D**) were normal. After the loss of follow-up for 5 years, acute decompensated heart failure developed as his chest X-ray revealed cardiomegaly and lung edema (**B**), and his EKG revealed atrial fibrillation (**E**). After the proper treatment, his symptoms subsided, and his chest X-ray showed improvement (**C**).

A 49-year-old man visited our out-patient-department (OPD) about an enlargement of the neck mass. He denied any symptoms, even having had goiter for years. He has a sister who also has a goiter and has been diagnosed to have thyroid dysfunction. Thyroid sonography revealed bilateral muti-nodular goiter. Laboratory data showed mildly elevated free T4, TSH, anti-TPO, and normal TSH receptor Ab. Specifically, data indicated 2.99 ng/dl (0.2–2) of free T4, 4.22 µIU/mL (0.4–4) of TSH, 11.49% (<15) of TSH receptor Ab, and 101 IU/mL (<35) of anti-TPO. According to the data, TSH-secreting pituitary tumor was suspected. The pituitary MRI showed suspicious microadenoma, but the TRH stimulation test was normal. Other axes of pituitary functions were normal. Under the impression of thyroid hormone resistance syndrome, a genetic study was advised, but the patient refused. The patient did not come for a follow-up until 5 years later. An episode of shortness of breath brought him to our ER. The electrocardiogram (EKG) revealed atrial fibrillation (Figure 1E), and the chest X-ray showed cardiomegaly and pulmonary edema (Figure 1B). Under the impression of acute decompensated heart failure, he was admitted. The echocardiogram revealed dilated left atrium (LA), left ventricle (LV), and symmetrical LV hypertrophy. A serial test, including a cardiopulmonary exercise test and Thallium 201 cardiac perfusion scan, was performed, and no evidence of coronary arterial disease was found. His symptoms improved after medical treatment with carbimazole 10 mg bid and propranolol 10 mg bid, and the subsequent chest X-ray showed improvement (Figure 1C). By the time of this acute episode, he agreed to take a genetic test. A heterozygous missense mutation c.959G>T (p.R320L) of the *THRβ* gene (NM_001252634) was noted.

### 2.4. Affected Kindred

**Figure 2:**
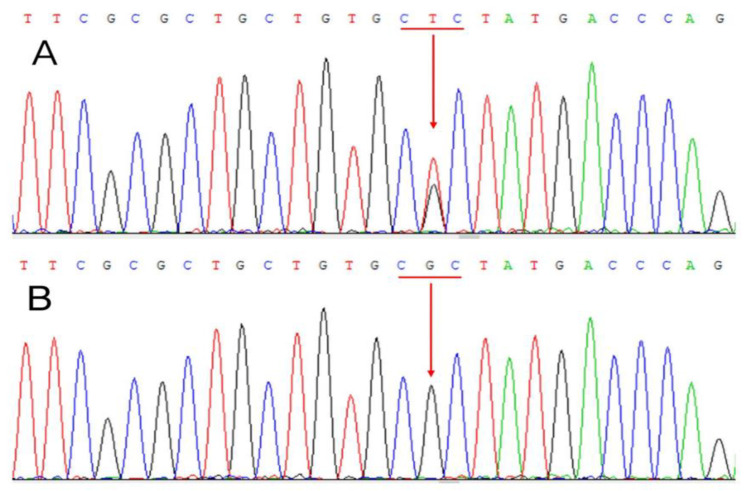
Sanger sequencing of the *THRβ* gene of the affected kindred. A heterozygous missense mutation c.959G>T of the *THRβ* gene was revealed in (**A**). This mutation leads to an R320L substitution in the *THRβ* protein. (**B**) shows wild type.

The only son of the proband, a 19-year-old man who was a university student, visited our OPD with symptoms of anxiety, palpitation, hand tremor, and insomnia without noticeable weight loss. He had average height and weight, and his mentality was normal. He exhibited mildly elevated free T4 levels, with normal amounts of TSH and TSH receptor Ab (TSHR Ab). Laboratory data showed 2.57 ng/dL (0.2–2) of free T4, 1.52 µIU/mL (0.4–4) of TSH, and 1.91% (<15) of TSHR Ab. Carbimazole 10 mg bid and propranolol 10 mg bid were given, and symptoms improved. Further studies were conducted at our OPD, including thyroid ultrasound, pituitary function tests, pituitary MRI, electrocardiogram (EKG), 24-h Holter EKG, and echocardiogram. Only bilateral multinodular goiter was noted in the thyroid ultrasonography. The pituitary function tests and MRI imaging were normal. The heart examinations were normal in both rhythm and function. The genetic study revealed a heterozygous mutation c.959G>T, (p.R320L) of the *THRβ* gene (NM_001252634) (Figure 2). During his follow-up period, we tried to discontinue carbimazole with a higher dose of propranolol, but free T4 kept going up to 4.65 ng/dL with worsening symptoms. We had to resume carbimazole and continue titrating it according to free T4 levels and the symptoms. A follow-up echocardiogram one year later revealed normal LV function.

## 3. Discussion

For patients with persistent elevation of circulating free T3 and free T4 levels, the differential diagnosis, in addition to RTH, was TSH-secreting pituitary adenoma. In our proband, the first brain MRI showed suspicious pituitary microadenoma. In order to rule out TSH-secreting pituitary adenomas, the TRH stimulation test was done. The response was normal, with TSH increasing more than 50% after TRH stimulation, and the diagnosis of TSH-secreting pituitary adenomas was excluded [6].

The *THRβ* gene with heterogeneous mutation c.959 G>T (p.R320L) is exceedingly rare. There had been only two cases with the same mutation reported in one study, and mutation in this region decreased the affinity between thyroid hormone receptor β and triiodothyronine, T3 [7]. In the past, the clinical presentations of RTH were proposed to classify RTH, such as generalized resistance to thyroid hormone (GRTH), with which patients were usually asymptomatic, and pituitary resistance to thyroid hormone (PRTH), with which patients may have thyrotoxic symptoms. In previous studies of the two cases with c.959 G>T (p.R320L) mutation of the *THRβ* gene, one was classified in GRTH, and the other was in PRTH [7]. In fact, the clinical manifestations of RTH vary widely, even in members of the same family who bear the same mutation. Nowadays, the terms GRTH and PRTH are no longer used to classify RTH. In addition to R320L, mutations at codon 320 of the *THRβ* gene with different nucleotides have also been reported, such as R320H, R320C, and R320P [7,8,9,10]. Manifestations of those other codons 320 mutations were not all clearly recorded in the previous pieces of literature, and the age of these patients if mentioned, were all in the 30 s. Among them, a 31-year-old Japanese male with R320P mutation [9], such as our proband, had a history of atrial fibrillation status post cardiac catheter ablation when he was 21 and 25 years old. We did not know if the cardiac manifestation was related to gender or race, given such limited data. The distribution of different subtypes of thyroid hormone receptors is tissue specific. In the myocardium, *THRα* is predominant. Thus, patients with *THRβ* mutation are likely to have cardiac symptoms. It was observed that cardiovascular features were not corelated with the genotype and progressed with aging [11,12]. Both our proband and affected kindred had cardiac symptoms. The son of the proband had only palpitations at his first visit. Our proband developed atrial fibrillation and cardiomegaly with acute decompensation heart failure after 5 years of no follow-up. In previous studies, patients with *THRβ* were given L-T_3_ and/or propranolol or were not given any medication. In our cases, the cardiac symptoms could only be relieved by the combination of carbimazole and propranolol. Electrocardiogram and echocardiography every 2–3 years were recommended in symptomatic patients [13]. However, in our experience, regular electrocardiograms and echocardiography should be performed in older patients without any symptoms. Family screening may help us treat patients as early as possible to prevent severe cardiac complications.

## 4. Conclusions

RTH is a rare disease with impaired tissue responsiveness to TH. We report a family with a rare heterozygous point mutation, c.959G>T, (p.R320L) of the *THRβ* gene. The proband developed atrial fibrillation and life-threatening heart failure with pulmonary edema, which was quite different from previously reported *THRβ* gene mutations. Considering the rareness of RTH and the heterogeneity of its phenotypes, our report allows for a better understanding of the manifestation and management of patients with RTH and *THRβ* gene mutation.

## Data Availability

Data are available upon request to authors.

## References

[1] Persani L., Campi I. (2019). Syndromes of Resistance to Thyroid Hormone Action. Genetics of Endocrine Diseases and Syndromes.

[2] Minakhina S., Bansal S., Zhang A., Brotherton M., Janodia R., De Oliveira V., Tadepalli S., Wondisford F.E. (2020). A Direct Comparison of Thyroid Hormone Receptor Protein Levels in Mice Provides Unexpected Insights into Thyroid Hormone Action. Thyroid.

[3] Refetoff S., Bassett J.H., Beck-Peccoz P., Bernal J., Brent G., Chatterjee K., De Groot L.J., Dumitrescu A.M., Jameson J.L., Kopp P.A. (2014). Classification and proposed nomenclature for inherited defects of thyroid hormone action, cell transport, and metabolism. Thyroid.

[4] Brucker-Davis F., Skarulis M.C., Grace M.B., Benichou J., Hauser P., Wiggs E., Weintraub B.D. (1995). Genetic and clinical features of 42 kindreds with resistance to thyroid hormone. The National Institutes of Health Prospective Study. Ann. Intern. Med..

[5] Sakurai A., Miyamoto T., Refetoff S., DeGroot L.J. (1990). Dominant negative transcriptional regulation by a mutant thyroid hormone receptor-beta in a family with generalized resistance to thyroid hormone. Mol. Endocrinol..

[6] Tjörnstrand A., Nyström H.F. (2017). DIAGNOSIS OF ENDOCRINE DISEASE: Diagnostic approach to TSH-producing pituitary adenoma. Eur. J. Endocrinol..

[7] Adams M., Matthews C., Collingwood T.N., Tone Y., Beck-Peccoz P., Chatterjee K.K. (1994). Genetic analysis of 29 kindreds with generalized and pituitary resistance to thyroid hormone. Identification of thirteen novel mutations in the thyroid hormone receptor beta gene. J. Clin. Investig..

[8] Cannarella R., Musmeci M., Garofalo V., Timpanaro T.A., Leone G., Caruso M., Maltese P.E., Condorelli R.A., Vignera S.L., Calogero A.E. (2022). Resistance to Thyroid Hormones: A Case-Series Study. Int. J. Mol. Sci..

[9] Kimura T., Hayashi Y., Tsukamoto Y., Okamoto Y. (2018). The Mutant Thyroid Hormone Receptor Beta R320P Causes Syndrome of Resistance to Thyroid Hormone. Case Rep. Endocrinol..

[10] Weiss R.E., Weinberg M., Refetoff S. (1993). Identical mutations in unrelated families with generalized resistance to thyroid hormone occur in cytosine-guanine-rich areas of the thyroid hormone receptor beta gene. Analysis of 15 families. J. Clin. Investig..

[11] Illouz F., Briet C., Mirebeau-Prunier D., Bouhours-Nouet N., Coutant R., Sibilia P., Rodien P. (2021). Cardiac complications of thyroid hormone resistance syndromes. Ann. Endocrinol..

[12] Kahaly G.J., Matthews C.H., Mohr-Kahaly S., Richards C.A., Chatterjee V.K. (2002). Cardiac involvement in thyroid hormone resistance. J. Clin. Endocrinol. Metab..

[13] Pulcrano M., Palmieri E.A., Mannavola D., Ciulla M., Campi I., Covelli D., Lombardi G., Biondi B., Beck-Peccoz P. (2009). Impact of resistance to thyroid hormone on the cardiovascular system in adults. J. Clin. Endocrinol. Metab..

